# Biomarkers for predicting the severity of spinal cord injury by proteomic analysis

**DOI:** 10.3389/fnmol.2023.1153230

**Published:** 2023-12-13

**Authors:** Liangfeng Wei, Yubei Huang, Yehuang Chen, Jianwu Wu, Kaiqin Chen, Zhaocong Zheng, Shousen Wang, Liang Xue

**Affiliations:** ^1^Fuzong Clinical Medical College of Fujian Medical University (900TH Hospital), Fuzhou, China; ^2^Department of Neurosurgery, Fuding Hospital, Fujian University of Traditional Chinese Medicine, Fuding, China; ^3^Department of Neurosurgery, Xiang’an Hospital of Xiamen University, Xiamen, China

**Keywords:** spinal cord injury, spinal cord injury rat model, behavior assessment, pathological changes, proteomic analysis, different extent of spinal cord damage

## Abstract

**Purpose:**

Currently, there is a shortage of the protein biomarkers for classifying spinal cord injury (SCI) severity. We attempted to explore the candidate biomarkers for predicting SCI severity.

**Methods:**

SCI rat models with mild, moderate, and severe injury were constructed with an electro-mechanic impactor. The behavior assessment and pathological examinations were conducted before and after SCI. Then, quantitative liquid chromatography-mass spectrometry (LC-MS/MS) was performed in spinal cord tissues with different extents of injury. The differentially expressed proteins (DEPs) in SCI relative to controls were identified, followed by Mfuzz clustering, function enrichment analysis, and protein-protein interaction (PPI) network construction. The differential changes of candidate proteins were validated by using a parallel reaction monitoring (PRM) assay.

**Results:**

After SCI modeling, the motor function and mechanical pain sensitivity of SCI rats were impaired, dependent on the severity of the injury. A total of 154 DEPs overlapped in the mild, moderate, and severe SCI groups, among which 82 proteins were classified in clusters 1, 2, 3, 5, and 6 with similar expression patterns at different extents of injury. DEPs were closely related to inflammatory response and significantly enriched in the IL-17 signaling pathway. PPI network showed that Fgg (Fibrinogen gamma chain), Fga (Fibrinogen alpha chain), Serpinc1 (Antithrombin-III), and Fgb (Fibrinogen beta chain) in cluster 1 were significant nodes with the largest degrees. The upregulation of the significant nodes in SCI samples was validated by PRM.

**Conclusion:**

Fgg, Fga, and Fgb may be the putative biomarkers for assessing the extent of SCI.

## Introduction

Spinal cord injury (SCI) as a common complication of spine injury, is associated with permanent loss of motor and sensory function (Hamid et al., 2018). Traumatic SCI has led to tremendous social and financial costs (van der Scheer et al., 2018). The burden derived from SCI is increasing with rising incidence worldwide (Selvarajah et al., 2014). It is reported that there are an estimated 250,000 to 500,000 cases of SCI each year and the lives of two to three million individuals are tremendously influenced by SCI worldwide (Quadri et al., 2020). Despite the advances in the treatment methods for SCI, the mortality of SCI patients remains high with 3.1–22.2% in developed regions and 1.4–20.0% in non-developed regions (Kang et al., 2018). Currently, there is no cure for SCI. Thus, the early diagnosis and severity assessment of SCI are necessary.

The currently existing non-invasive conventional SCI evaluation systems include the American Spinal Injury Association impairment scale (AIS) and quantitative magnetic resonance imaging (MRI). AIS scale has become the gold standard for SCI assessment, which is helpful in determining the complete and incomplete injury and guiding further treatment (Roberts et al., 2017). Despite the proven benefit of the AIS scale, it falls behind in distinguishing subjects with similar AIS scores in other respects. MRI is widely used in determining the injury location and extent of intramedullary in SCI with edema and hemorrhage (Seif et al., 2019). However, MRI is limited in detecting microstructure disruption in spinal cord (Cohen-Adad, 2018).

Emerging evidence indicates that biomarkers exert potential in classifying injury severity of SCI (Dalkilic et al., 2018; Ogurcov et al., 2021). It is reported that the levels of the biomarkers in serum and cerebrospinal fluid, such as GFAP, NSE, and MCP-1 were increased in a time-dependent manner following SCI and related to SCI severity (Leister et al., 2020). The Tau protein concentration in serum and cerebrospinal fluid is positively correlated with SCI severity and shows prognostic implications (Tang et al., 2019). A recent pilot study has indicated that blood serum cytokines such as CXCL5, CCL11, and IL10 can be used as candidate biomarkers for stratifying SCI severity (Ogurcov et al., 2021). However, the protein biomarkers for predicting SCI severity may be insufficient.

With the advances in proteomic techniques, the study of differential proteome in SCI has been achieved. Therefore, in our study, we performed proteomic analysis of spinal cord tissues of SCI animal models. The differential expressed proteins (DEPs) in mild SCI, moderate SCI, and severe SCI compared with normal controls were identified, followed by functional annotation and protein-protein interaction (PPI) network analysis. We attempted to explore the clinically useful biomarkers for predicting injury severity following SCI.

## Materials and methods

### Animals and groups

A total of 40 specific-pathogen-free (SPF) healthy Sprague Dawley (SD) rats (weight range: 180–220 g) were provided by the 900TH Hospital. The study was approved by the Animal Care and Use Committee of the 900TH Hospital and all the animal-related procedures complied with the guide for the care and use of animals. Animals were kept in cages under 24–26°C and 12/12h light-dark cycles with food at freedom. After 1 week of acclimatization, the rats were randomly divided into four groups, including the control group (*n* = 10), mild SCI group (*n* = 10), moderate SCI group (*n* = 10), and severe SCI group (*n* = 10).

### Spinal cord injury rat model construction

Rats in SCI groups were fasted for 12 h and then anesthetized with 3% pentobarbital sodium (30 mg/kg) prepared for surgery. After depilation of the lumbar and back region, 0.5 cm width dura mater at thoracic vertebra T10 was exposed. Then, rats were subjected to a contusion injury by a 68099II Brain and Spinal Cord Impactor (RWD Life Science, Shenzhen, Guangdong, China). The impact center point was DV (dorsoventral) 2.0, 4.0, and 6.0 mm in the mild, moderate, and severe SCI groups, respectively. The impact velocity was 2.5 m/s and the duration was 0.5 s. In the control group, there was no treatment for the rats. After injury, rats were subcutaneously injected with 5 mL lactated Ringer’s solution for water replenishment and intramuscularly injected with gentamicin (1.2 ml/kg) once daily for consecutive 7 days to prevent post-injury infection. In addition, bladders were expressed manually 3–4 times a day. All the rats were allowed to recover in SPF cages with access to food and water freely.

### Basso, beattie, bresnahan (BBB) locomotor rating scale

After injury, the hindlimb behaviors of rats were assessed by the BBB scale (Basso et al., 1995). The hindlimb movements were scored 0 to 21 points, which represented the function of hindlimb movement. The movement functions of rats were evaluated 1 day before injury and 1, 3, 7, 14, and 21 days post-injury by double-blind method.

### Mechanical pain threshold measurement

The mechanical pain threshold was evaluated in rats before and after SCI by the Von Frey filament test. Animals were habituated in wire mesh chambers for 30 min before testing and then filaments were applied as the controlled force to stimulate the finger web of rats for 8 s. The pain threshold was defined when rats elicited leg retraction, escape, or licking. The Von Frey filament test was performed twice at each time point of each rat with a 1 min interval. The mean value was calculated as the mechanical pain threshold.

### Hematoxylin and eosin (HE) staining

At 7 days after injury, rats in each group (*n* = 3) were anesthetized and transcardially perfused with 100 mL 0.1 mol/L cacodylate buffer, followed by 250 mL 4% paraformaldehyde. Spinal cords (about 1 cm to the front and back of the injury site) were isolated from the spinal column and dehydrated in 20 and 30% sucrose. Then, the tissues were sectioned at 4 μm thickness and stained for HE. The remaining tissues were stored in liquid nitrogen at −80°C.

### Protein sample preparation

Spinal cord tissues (*n* = 3 per group) were ground in liquid nitrogen, followed by lysis with Lysis buffer containing 8 M urea, 1% protease inhibitor, and 1% phosphatase inhibitor at a volume ratio of 1:4. Protein supernatants were obtained by centrifugation at 12,000 × *g* for 10 min at 4°C. The protein concentration was measured by a BCA assay kit (Beyotime, Shanghai, China). Then, the protein solution was incubated with 5 mM DTT at 56°C for 30 min and 11 mM Iodoacetamide for 15 min in the dark at room temperature. After the urea was diluted to 2 M in the protein solution, trypsin was added at a mass ratio of 1:50 (trypsin: protein) at 37°C overnight and a mass ratio of 1:100 (trypsin: protein) for a further 4 h. Subsequently, the peptide fragments were labeled with tandem mass tags (TMT) 6plex kit (Thermo Fisher Scientific Inc., Waltham, MA, USA) adherence to the manufacturer’s protocol.

### LC-MS/MS (tandem mass spectrometry) analysis

The peptide fragments were separated by ultra-high-performance liquid chromatography (UHPLC) EASY-nLC 1000 system and then subjected to NSI ionization source followed by tandem MS/MS in Q ExactiveTM Plus. The raw MS data were retrieved by Maxquant engine version 1.5.2.8 (Matrix Science, London, UK). The tandem mass spectra were searched against the Proteome Rat database (29955 sequences). The proteins were identified with FDR ≤ 0.01 and quantified by Proteome Discoverer 1.4 software.

### Bioinformatic analysis

The differentially expressed proteins (DEPs) were identified in mild, moderate, and severe SCI groups compared with the control group separately by Fisher’s exact test with the cutoff value of *p*-value < 0.05 and fold change (FC) >1.3 or < 1/1.3. The overlapped DEPs in mild, moderate, and severe SCI groups were obtained by a Venn diagram online tool. The DEPs were further screened by soft clustering with the implementation of the Mfuzz package (Kumar and Futschik, 2007) based on a fuzzy c-means algorithm. DEPs with similar expression patterns were classified into three GO categories: biological process (BP), cellular component (CC), and molecular function (MF), as we; as Kyoto Encyclopedia of Genes and Genomes (KEGG) pathways. The enrichment of GO terms and pathways was evaluated by a two-tailed Fisher’s test. *P* < 0.05 was considered significant. Besides, the interactions between proteins were retrieved from STRING database version 11.0. Protein pairs with confidence score ≥0.7 were collected for interaction network construction by networkD3 package in R.

### Targeted proteomics analysis

The differential expression of four protein nodes of interest in the PPI network was validated by PRM analysis. One or two unique peptides of each target protein were selected from the discovery measurements. A total of 6 protein samples (1 mild SCI vs. 1 Con; 1 moderated SCI vs. 1 Con; 1 severe SCI vs. 1 Con) were digested and injected into the LC-MS/MS system. PRM assay was performed in a data-independent procedure, with AGC at 3E6, maximum IT of 50 ms for one scan, and AGC target to IE5, maximum IT of 250 ms for the following 20 MS/MS scans. The isolation window was set as 1.6 m/z. For PRM analysis, the MS data were processed by Skyline software version 21.1.

### Statistical analysis

The statistical analysis was achieved by SPSS 26.0 software. The measurement data in accordance with normal distribution were expressed as mean ± SD. The multi-group comparison was conducted by one-way ANOVA and pairwise comparison was performed by Bonferroni’s test. The difference with *p* < 0.05 was considered significant.

## Results

### Behavioral testing

**FIGURE 1:**
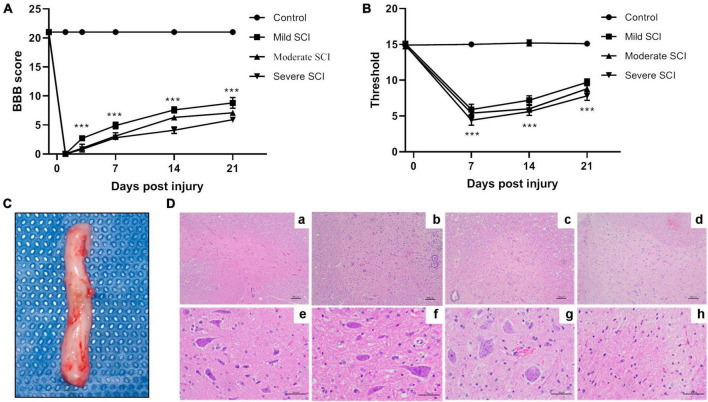
The behavior and pathological assessment of SCI rat models. The mild, moderate, and severe injury of spinal cords in rats were induced by an electro-mechanic impactor. **(A)** Time course of function recovery assessed by Basso, Beattie Bresnahan locomotor rating (BBB) scale. At 1 day before injury and 1, 3, 7, 14, and 21 days after SCI, BBB scores were recorded in SCI and control groups. ***p < 0.001. **(B)** Time course of mechanical pain threshold evaluated by Von Frey filament test. Von Frey test was performed 1 day before SCI and 7, 14, and 21 days after SCI in different groups, respectively. ***p < 0.001. **(C)** Spinal cord tissues (about 1 cm to the front and back of the injury site). The arrow indicates the SCI lesion site. **(D)** Representative image of HE staining for spinal cord tissues. a–d, magnification × 100. e–h, magnification × 400.

To determine the severity of injury in SCI rat models, the behaviors of rats were evaluated by BBB scores and mechanical pain threshold. As depicted in Figure 1A, rats in the control group elicited expected levels of BBB score (21.00 ± 0.00). The BBB score was reduced to zero at day 1 post-injury in SCI groups, while gradually increasing with a peak of 8.80 ± 0.92, 7.10 ± 0.32, and 5.90 ± 0.32 in the mild, moderate, and severe SCI groups at day 21 post-injury, respectively. The BBB score was significantly altered among SCI groups beyond 3 days post-injury (*p* < 0.001). Figure 1B illustrated that the mechanical pain threshold of rats in SCI groups was significantly decreased at different time points post-injury relative to controls (*p* < 0.001). SCI rats exhibited the most obvious reduction of mechanical pain threshold at day 7 post-injury compared with controls (*p* < 0.001). The threshold to mechanical pain sensitivity was lowest in the severe SCI group at different time points post-injury, compared with other SCI groups (*p* < 0.001). These suggested that the mechanical pain threshold of SCI rats was reduced, dependent on the severity of the injury.

### Pathological changes

At day 7 post-injury, spinal cords (about 1 cm to the front and back of the injury site) were isolated from the spinal column for HE staining (Figure 1C). HE staining showed a clear and intact structure of white and gray matter and clear borders of the spinal cord tissues in the control group. Meanwhile, the neurons exhibited uniform distribution, normal morphology, and intact cell membrane in the control group. Rats in mild SCI exhibited loose structure, glial infiltration, and cell swelling in gray matter. In the moderate group, there were necrotic tissues, blurred borders, neuron loss, and glial proliferation. The injured spinal cord in the severe group showed severe loss of gray and white matter, few neurons, and amounts of glial infiltration in remaining tissues (Figure 1D).

### DEP identification

**FIGURE 2:**
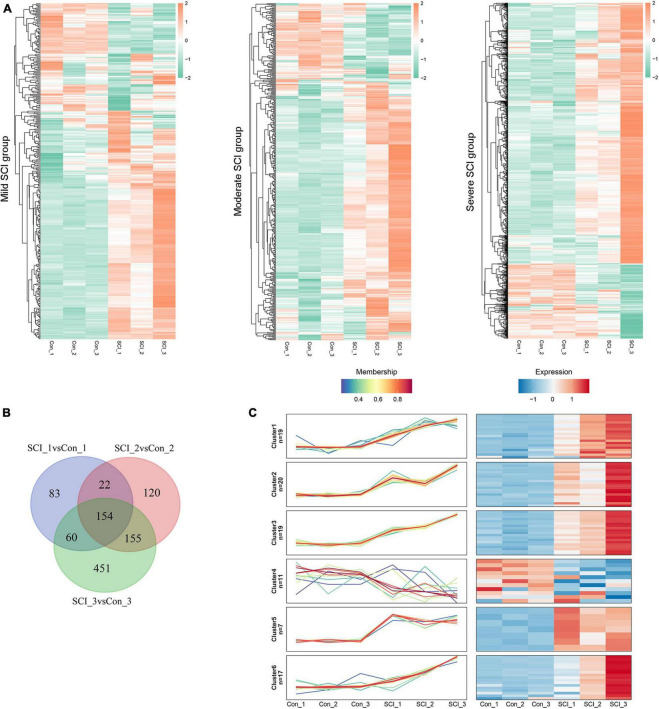
Differentially expressed proteins (DEPs) identification. **(A)** Heatmap for DEPs in mild, moderate, and severe SCI groups, compared with controls. Red, up-regulated proteins; green, down-regulated proteins. **(B)** Venn diagram analysis for overlapped DEPs in mild, moderate, and severe SCI groups. **(C)** Profiles of the six clusters obtained from the Mfuzz analysis. Red, up-regulated proteins; blue, down-regulated proteins. SCI1, SCI2, and SCI3 indicate mild, moderate, and severe group, respectively.

Compared with controls, we identified 319 DEPs (217 up-regulated and 102 down-regulated) in the mild SCI group, 457 (348 up-regulated and 109 down-regulated) in the moderate SCI group, and 820 (637 up-regulated and 183 down-regulated) in severe SCI group. The heatmap of DEPs (Figure 2A) displayed that the expression profiles of DEPs could distinguish control and SCI samples clearly, determining the significance of DEPs. By Venn analysis, there were 154 overlapped DEPs among mild, moderate, and severe SCI groups (Figure 2B). Then, the Mfuzz package was employed to detect the stage-related expression profiling of the candidate proteins in SCI progression (Figure 2C). A total of 93 eligible DEPs were classified into six clusters and each cluster exhibited a specific expression pattern of proteins. The proteins in cluster 1 (19 proteins), cluster 2 (20 proteins), cluster 3 (19 proteins), cluster 5 (7 proteins), and cluster 6 (17 proteins) showed similar expression trends separately. The expression levels of cluster 1, cluster 3, and cluster 6 proteins increased from the mild SCI group to the severe SCI group. The cluster 2 and cluster 5 protein expression increased at the mild stage, decreased at the moderate stage, and then elevated at the severe stage. Thus, the 82 DEPs were screened out for further analysis.

### Function enrichment analysis

**FIGURE 3:**
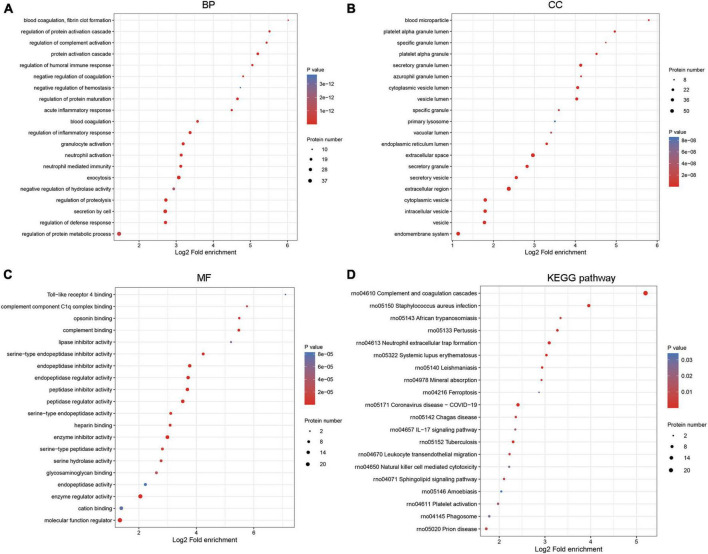
Functional enrichment analysis for DEPs. The biological function was analyzed by GO annotation in three GO categories, including **(A)** biological process (BP), **(B)** cellular component (CC), and **(C)** molecular function (MF). **(D)** The significant pathways related to DEPs were predicted by the Kyoto Encyclopedia of Genes and Genomes (KEGG) database.

The biological function of 82 DEPs was dissected based on GO annotation and KEGG pathway enrichment analysis. Results depicted that DEPs were closely associated with the regulation of inflammatory response, acute inflammatory response related BP, extracellular space, secretory granule-related CC, and peptidase regulator activity, complement binding-related MF (Figures 3A–C). The pathways of Leukocyte transendothelial migration, IL-17 signaling pathway, and Natural killer cell-mediated cytotoxicity were significantly enriched by target DEPs (Figure 3D).

### Protein-protein interaction (PPI) network

**FIGURE 4:**
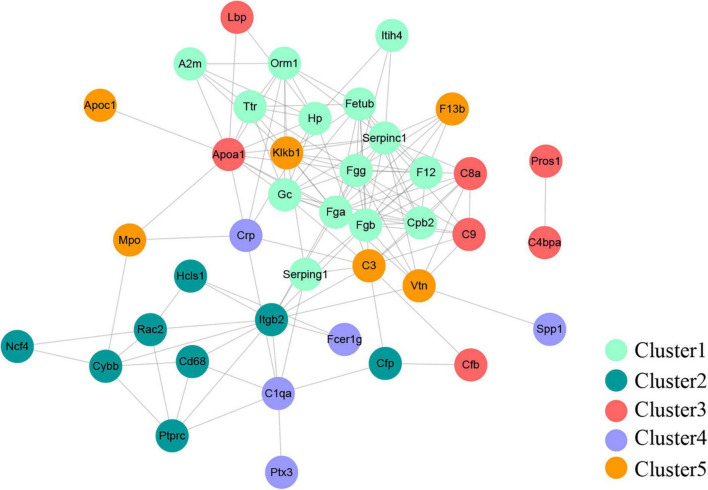
Protein-protein interaction network. The PPI network was comprised of 133 edges and 82 nodes.

A PPI network with 133 edges connecting with 82 nodes was constructed (Figure 4). The topological characteristics of the PPI network were analyzed. The nodes with large degrees were mined, such as Fgg (Fibrinogen gamma chain, degree = 16), Fga (Fibrinogen alpha chain, degree = 15), Serpinc1 (Antithrombin-III, degree = 15), and Fgb (Fibrinogen beta chain, degree = 13) (Table 1). The proteins with large degrees played key roles in PPI network construction and exerted the potential as the biomarkers for SCI.

**TABLE 1:** Top 15 significant nodes with largest degrees in PPI network.

Cluster	Protein accession	Protein description	Gene name	Degree
Cluster1	P02680	Fibrinogen gamma chain	Fgg	16
Cluster1	Q7TQ70	Fibrinogen alpha chain	Fga	15
Cluster1	Q5M7T5	Antithrombin-III	Serpinc1	15
Cluster1	P14480	Fibrinogen beta chain	Fgb	13
Cluster3	P04639	Apolipoprotein A-I	Apoa1	13
Cluster2	B2RYB8	Integrin beta	Itgb2	12
Cluster6	M0RBF1	C3-beta-c	C3	11
Cluster1	P04276	Vitamin D-binding protein	Gc	11
Cluster1	Q9QX79	Fetuin-B	Fetub	11
Cluster1	Q9EQV9	Carboxypeptidase B2	Cpb2	10
Cluster1	P06866	Haptoglobin	Hp	9
Cluster1	P02764	Alpha-1-acid glycoprotein	Orm1	9
Cluster1	D3ZTE0	Coagulation factor XII	F12	9
Cluster6	P14272	Plasma kallikrein	Klkb1	9
Cluster3	D3ZWD6	Complement C8 alpha chain	C8a	8

### Validation of DEPs for SCI

**FIGURE 5:**
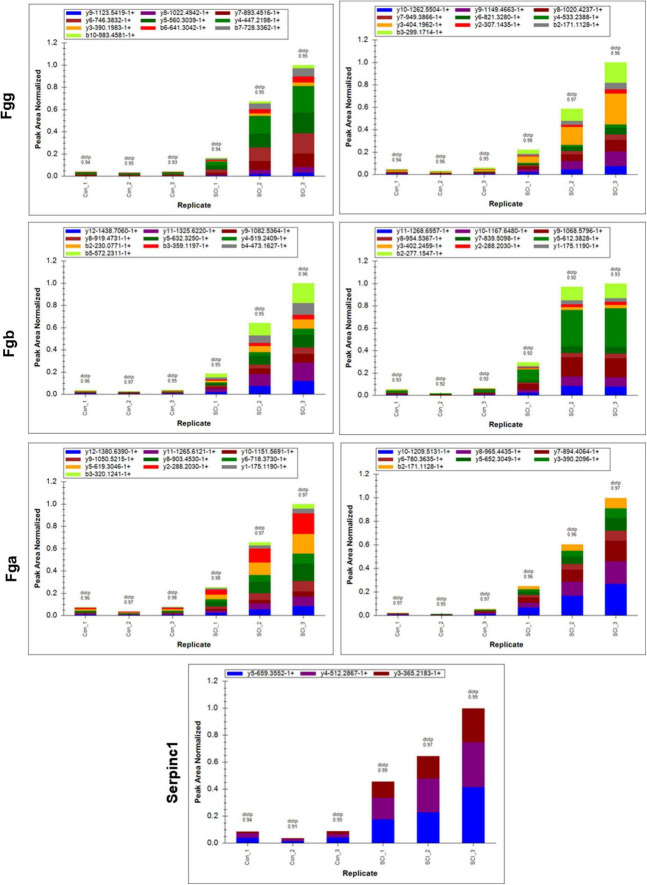
Peak area of target peptide in all samples by PRM assay. The target peptide sequence for Fgg is right: EGFGHLSPTGTTEFWLGNEK, **left**: VAQLEAQCQEPCK; for Fgb is **right**: DNENVINEYSSILEDQK, **left**: LYIDETVNDNIPLNLR; for Fga is **right**: GDFANANNFDNTFGQVSEDLR, **left**: GLIDEANQEFTNR; for Serpinc1 is TSDQIHFFFAK.

The differential changes of proteins of interest in SCI were validated by using PRM on the same samples from the discovery sets. Results showed that the four significant nodes in the PPI network (Fgg, Fga, Serpinc1, Fgb) were significantly overexpressed in SCIs vs. controls (Table 2 and Figure 5), which were consistent with the bioinformatic analysis.

**TABLE 2:** Parallel reaction monitoring (PRM) verified upregulated proteins in SCI compared to controls.

Protein accession	Protein description	Gene name	Peptide sequence	SCI_1/Con_1 Ratio PRM	SCI_2/Con_2 Ratio PRM	SCI_3/Con_3 Ratio PRM	*P*-value
P02680	Fibrinogen gamma chain OS = Rattus norvegicus OX = 10116 GN = Fgg PE = 1 SV = 3	Fgg	VAQLEAQCQEPCK; EGFGHLSPTGTTEFWLGNEK	4.01	18.35	18.9	*p* < 0.05
P14480	Fibrinogen beta chain OS = Rattus norvegicus OX = 10116 GN = Fgb PE = 1 SV = 4	Fgb	DNENVINEYSSILEDQK; LYIDETVNDNIPLNLR	5.03	30.57	18.71	*p* < 0.05
Q5M7T5	Antithrombin-III OS = Rattus norvegicus OX = 10116 GN = Serpinc1 PE = 1 SV = 1	Serpinc1	TSDQIHFFFAK	5.18	16.65	11.28	*p* < 0.05
Q7TQ70	Fibrinogen alpha chain OS = Rattus norvegicus OX = 10116 GN = Fga PE = 1 SV = 1	Fga	GLIDEANQEFTNR; GDFANANNFDNTFGQVSEDLR	5.13	23.99	14.88	*p* < 0.05

## Discussion

Spinal cord injury as one major cause of disability, has been emphasized for its increasing incidence rate (Selvarajah et al., 2014). Despite the advances in medical, surgical, and rehabilitative care, the lifespan and life quality of patients with SCI remains to be improved. The severity of the initial injury of SCI exerted a critical role in determining the secondary response and guiding the corresponding interventions. However, the assessment of injury severity following SCI remains to be a challenge.

The rat model of SCI as a favored mammalian model is widely applied to investigate pathological events following injury. SCI rats can model human conditions with different types of injury, including complete and incomplete injuries of the spinal cord at different extents (Kjell and Olson, 2016). In the present study, SPF SD rats were used for SCI model construction, which were induced by a precisely controlled electro-mechanic impactor that allowed us to control the extent of injury (mild, moderate, and severe) in different cohorts of rats. Behavioral tests were applied to evaluate the symptoms developed in SCI rat models. BBB score is one of the available scoring systems to evaluate the loss and recovery of locomotor function (Basso et al., 1996). BBB score is demonstrated to be reliable and helpful in assessing the injury from moderate to severe (Lankhorst et al., 1999). In addition, sensory function is another important aspect to be monitored for the successful SCI model, which is widely measured by the von Frey test (Chaplan et al., 1994). Our results suggested that the motor function and mechanical pain sensitivity of SCI rats were significantly impaired depending on the severity of injury, in parallel with the different extents of pathological changes. These determined that SCIs with mild, moderate, and severe injury were successfully induced in different groups of rats.

The proteome is designed for observing the protein expression changes perturbed by disease or drugs (Anderson and Anderson, 1998). Currently, proteomic technology has been increasingly used for studying the proteomic expression patterns in control and SCI groups with the application of animal models or clinical samples (Moghieb et al., 2016; Streijger et al., 2017; Kwon et al., 2019). Kang et al. (2006) analyzed the differential proteomic profiling of injured spinal cord tissues after 24 h-SCI and found that DEPs were annotated in neural function. The study by Yan et al. (2010) described the dynamic changes in protein profiling of spinal cord tissues after 8 h, and 1, 3, and 5 days post-SCI, aiming to explore the progressive pathology of SCI. Ding et al. (2020) dissected DEPs at 1, 2, 3, and 8 weeks post-SCI by utilization of iTRAQ. This study identified 29 common DEPs, which could be associated with the SCI progression through stages. However, proteomic studies for exploring the injury-severity-related biomarkers are insufficient. Therefore, in the present study, we deeply excavate the DEPs in mild, moderate, and severe injured spinal cord tissues compared with controls in a preclinical rat model with the goal of our study to identify the candidate biomarkers for predicting the injury severity after SCI.

To explore the biomarkers related to SCI severity, we profiled the protein expression of spinal cord tissues in mild, moderate, and severe groups by LC-MS/MS analysis. Results suggested that there were a total of 154 overlapped proteins with differential expression in three groups. Through Mufzz cluster analysis, 82 DEPs were divided into clusters 1, 2, 3, 5, and 6. Proteins exhibited similar expression patterns in each cluster.

Function enrichment analysis showed that the clustered DEPs were annotated in the regulation of inflammatory response BP category. After SCI, the spinal cord elicits a pronounced inflammatory response to injury. Neuroinflammatory response plays a critical role in determining the secondary damage following mechanical damage in SCI (Rust and Kaiser, 2017). The neuroinflammatory response is mediated by various types of cells, such as microglia, neutrophils, and macrophages. Inflammatory response has been suggested as the therapeutic target for SCI to improve injury outcomes (Orr and Gensel, 2018). Besides, our data also indicated that the IL-17 signaling pathway was significantly enriched by DEPs in clusters. IL-17 as a critical pro-inflammatory factor, exerts a key role in promoting inflammatory response and neuropathic pain following SCI (Zong et al., 2014; Sun et al., 2023). Recent evidence has indicated that IL-17A inhibition contributes to ependymal cell proliferation and motor recovery post-SCI (Miyajima et al., 2021). As mentioned above, the DEPs screened in our study were significant for SCI progression and our results were credible.

Furthermore, the PPI network was constructed to mine the candidate biomarkers for SCI. Our data showed that Fgg, Fga, Serpinc1, and Fgb were the significant nodes in the PPI network. The genes of Fga, Fgb, and Fgg encode the Aα, Bβ, and γ chain of fibrinogen, a hexameric glycoprotein essential in hemostasis (Simurda et al., 2020). The study of Davalos and Akassoglou (2012) suggested that fibrinogen played a pro-inflammatory role in SCI and exerted a critical role in determining the extent of inflammation. Another evidence showed that fibrinogen was negatively associated with nerve function by inhibiting the differentiation of oligodendrocyte progenitor cells (Norris and Strickland, 2017). Zhang et al. (2016) suggested that fibrinogen level was increased in multiple sclerosis and neuromyelitis optica, which was closely associated with the severity of these diseases. In the present study, the DEPs of fibrinogen alpha, beta, and gamma chain were all clustered into cluster 1, in which the proteins exhibited increasing expression profiles from mild to severe SCI group. Above all, fibrinogen alpha, beta, and gamma chains had increased expressions in SCIs with all extents of injury and could be proposed as the biomarker for SCI severity. In addition, Antithrombin-III (ATIII) encoded by Serpinc1 is a serine protease inhibitor. Lu et al. (2017) revealed that ATIII could be supported as the potential treatment for kidney diseases for its strong anticoagulant and anti-inflammatory action. ATIII insufficiency was associated with the severity of renal ischemia/reperfusion injury (Wang et al., 2015). However, role of ATIII in SCI has not been fully understood. Furthermore, PRM assay validated that the Fgg, Fgb, Fga, and Serpinc 1 were all upregulated in SCIs with different degrees of severity, which was consistent with the MS discovery set. Fgg exerted increased expression dependent on severity extent, whereas Fgb, Serpinc1, and Fga showed the highest expression in the moderate SCI group, which might be caused by the small sample size in the PRM assay. Thus, further studies are warranted.

In conclusion, the proteins with differential expression between SCI and controls played a pivotal role in the development and progression of SCI. The significant nodes in the PPI network with increasing expression could be the candidate biomarkers for SCI severity, such as Fga, Fgb, and Fgg.

## Data availability statement

The raw data supporting the conclusions of this article will be made available by the authors, without undue reservation.

## Ethics statement

The animal studies were approved by the Animal Care and Use Committee of the 900TH Hospital. The studies were conducted in accordance with the local legislation and institutional requirements. Written informed consent was obtained from the owners for the participation of their animals in this study.

## Author contributions

LW carried out the studies, drafted the manuscript, and performed the statistical analysis. YH, YC, JW, and KC participated in collecting data. SW, ZZ, and LX critically reviewed the manuscript. All authors read and approved the final manuscript.
